# The Activation Pattern of Blood Leukocytes in Head and Neck Squamous Cell Carcinoma Is Correlated to Survival

**DOI:** 10.1371/journal.pone.0051120

**Published:** 2012-12-10

**Authors:** Camilla Rydberg Millrud, Anne Månsson Kvarnhammar, Rolf Uddman, Sven Björnsson, Kristian Riesbeck, Lars Olaf Cardell

**Affiliations:** 1 Division of ENT diseases, Department of Clinical Science, Intervention and Technology, Karolinska Institutet, Karolinska University Hospital, Stockholm, Sweden; 2 Department of Otorhinolaryngology, Lund University, Skåne University Hospital, Malmö, Sweden; 3 Clinical Chemistry, Department of Laboratory Medicine Malmö, Lund University, Skåne University Hospital, Malmö, Sweden; 4 Medical Microbiology, Department of Laboratory Medicine Malmö, Lund University, Skåne University Hospital, Malmö, Sweden; University of Oslo, Norway

## Abstract

Head and neck squamous cell carcinoma (HNSCC) is known to cause substantial immunosuppression. The present study was designed to characterize blood leukocyte activation in HNSCC and to investigate if the individual activation pattern could be related to tumor progress and survival. The leukocyte activation profile of HNSCC patients and healthy controls was assessed with flow cytometry. HNSCC patients displayed increased numbers of monocytes, neutrophils and total leukocytes as well as an enhanced neutrophil/lymphocyte ratio. In addition, patients had a higher percentage of CD69^+^, CD71^+^ and CD98^+^ T cell subsets and NK cells, and a reduced expression of L-selectin in CD14^high^CD16^+^ monocytes and neutrophils, when compared to controls. These changes could be correlated to both tumor burden and spread to lymph nodes. Among the cancer patients an increased neutrophil/lymphocyte ratio, a low neutrophil and CD14^high^ CD16^+^ monocyte activation state and an elevated CD4/CD8 ratio were related to poor survival. In contrast, a high percentage of CD98^+^ Th cells appeared to be associated with a better outcome. Taken together, the present data indicate that HNSCC causes activation of blood leukocytes and that the individual activation pattern can be linked to prognosis.

## Introduction

Head and neck squamous cell carcinoma (HNSCC) is aggressive in nature. It induces production of cytokines and growth factors that regulate the expression of genes controlling growth, survival, and chemosensitivity [1], [2]. Such dysregulation of the inflammatory response is believed to perpetuate the malignant phenotype. In addition, HNSCC tumors have the ability to produce immunosuppressive mediators that affect the immune function of the host. Local interactions between the tumor and infiltrating leukocytes are also suggested to cause immunological alterations, *e.g.* an increased number of activated T cells [1], [2], [3], [4], [5], [6]. However, reports concerning leukocyte activation are not unequivocal. Some authors have found reduced leukocyte numbers among HNSCC patients, whereas others have not [3], [4], [7]. The present study was designed to investigate the role of leukocytes and their activation in HNSCC. To this end, peripheral blood from newly diagnosed, still untreated patients was compared to blood obtained from healthy age- and gender-matched controls. An attempt to link these changes to tumor burden, lymphatic spread and survival was also made.

## Materials and Methods

### Ethical Statement

The study was approved by the Ethics Committees of Karolinska Institutet and Lund University and a written informed consent was obtained from all participants.

### Patients

In total, 20 patients (14 males and 6 females) diagnosed with HNSCC were sampled before initiation of treatment along with 20 healthy controls (12 males and 8 females). The median age of the patients was 69 years (range 52–87) and of the controls 70 years (range 51–89). The control individuals were closely matched to the cancer patients regarding age, gender, medication, smoking and alcohol consumption. Neither control subjects nor HNSCC patients had autoimmune disorders, ongoing immune modulating medication or a previous history of malignant diseases. The clinical tumor (T) and lymph node (N) classification of the cancer patients at the time of inclusion are shown in Table 1. The patients' different tumor locations were as follows: 4 epipharyngeal, 2 esophageal, 6 tonsillar, 2 gingival, 1 laryngeal, 3 hypopharyngeal, 1 tongue, and finally 1 dermal location.

**Table 1:** Clinical tumor (T) and lymph node (N) classification of the HNSCC patients.

N stage						*Total*
		0	1	2	3	
**T stage**	**1**	0	0	0	0	*0*
	**2**	3	0	5	0	*8*
	**3**	4	0	1	0	*5*
	**4**	3	0	2	1	*6*
	**X** 1	0	0	1	0	*1*
*Total*		*10*	*0*	*9*	*1*	*20*

1 x – Tumor size not determined.

### Blood sampling

Two blood samples were collected from each individual; 4 ml in a test tube containing EDTA for leukocyte differential count analysis performed on a Coulter® LH750/GenS cell counter (Beckman Coulter, Marseille, France), and another 4 ml in a tube containing buffered tri-sodium citrate for flow cytometry analysis. To ensure identical blood sampling the procedure was carried out in the morning after at least 30 min rest.

### Antibodies and reagents

The monoclonal antibody (mAb) combinations used for flow cytometry analysis are presented in the Table S1. The following mAbs detecting various surface antigens were purchased from Beckman Coulter: CD3-ECD (clone UCHT1), CD4- PCy5 (13B8.2), CD11c-RPE (BU15), CD14-PCy5 (RMO52), CD16-ECD (3G8), CD16-PCy5 (3G8), CD25-ECD (B1.49.9), CD56-PCy5 (N901), CD62L-RPE (DREG56), CD64-PCy5 (22), CD69-RPE (TP1-55-3), CD69-ECD (TP1-55-3), CD71-FITC (YDJ1.2.2), chemoattractant receptor-homologous molecule expressed on T helper (Th)2 cells (CRTH2)-RPE (BM16) and human leukocyte antigen (HLA)-DR-PCy5 (Immu357). CD98-FITC (44D7) was obtained from Serotec (Oxford, UK), whereas forkhead box p3 (Foxp3)-FITC (PCH101) and CD123-FITC (6H6) were from eBioscience (San Diego, CA). CD8-FITC (DK25) and CD14-FITC (TÜK4) were purchased from DakoCytomation (Glostrup, Denmark), while CD71-PCy5 (M-A712) and lineage cocktail (Lin; FITC-conjugated mAbs directed against CD3 (SK7), CD14 (M?P9), CD16 (3G8), CD19 (SJ25C1), CD20 (L27) and CD56 (NCAM16.2)) were from BD Bioscience (San Jose, CA). An RPE-conjugated mAb against blood dendritic cell antigen 2 (BDCA2; AC144) was from Miltenyi Biotec (Bergisch Gladbach, Germany). The following isotype controls were used: msIgG-FITC, msIgG1-RPE (P3) and msIgG1-PCy5 (P3) from eBioscience, and msIgG2b-ECD (MPC-11) from Beckman Coulter.

### Flow cytometry analysis

Blood from 20 HNSCC patients and 20 healthy controls was analyzed on a Coulter Epics XL flow cytometer (Beckman Coulter) and leukocytes were gated based on forward and side scatter properties (Figure S1). Events in the range 40,000–200,000 were collected depending on the occurrence of the investigated leukocyte population, and analyzed with Expo32 analysis software (Beckman Coulter). To ensure flow cytometric standardization, the voltage settings were updated daily using FlowSet calibration beads (Beckman Coulter). All mAbs were titrated before use, and staining intensity was controlled on a weekly basis. For extracellular staining, 50 µl blood was incubated with mAbs for 20 min. Erythrocytes were lyzed with 600 µl 0.1% (v/v) formic acid for 3–5 seconds and the ionic strength was rendered iso-osmotic by addition of 280 µl 51 mM Na_2_CO_3_, 0.20 M Na_2_SO_4_ and 0.22 M NaCl. Intracellular staining of Foxp3 was performed using the IntraPrep™ Permeabilization Reagent Kit, according to instructions of the manufacturer (Beckman Coulter). For both extra- and intracellular staining, cells were washed in PBS and resuspended in PBS containing 1% formaldehyde prior to analysis.

### Statistics

Statistical analysis was performed using GraphPad Prism 5 (GraphPad Software, San Diego, CA). For normally distributed data statistical analysis was determined using unpaired Student's *t*-test, with Welch correction if the variances were non-homogenous. The nonparametric Mann Whitney test was used for not normally distributed unpaired data. *n* is equal to the number of independent donors and *p*-values≤0.05 were considered statistically significant. The survival function from life-time data was estimated using Kaplan-Meier analysis and a log rank test was utilized to examine the significance of the difference of the survival distribution between the groups.

## Results

**Figure 1:**
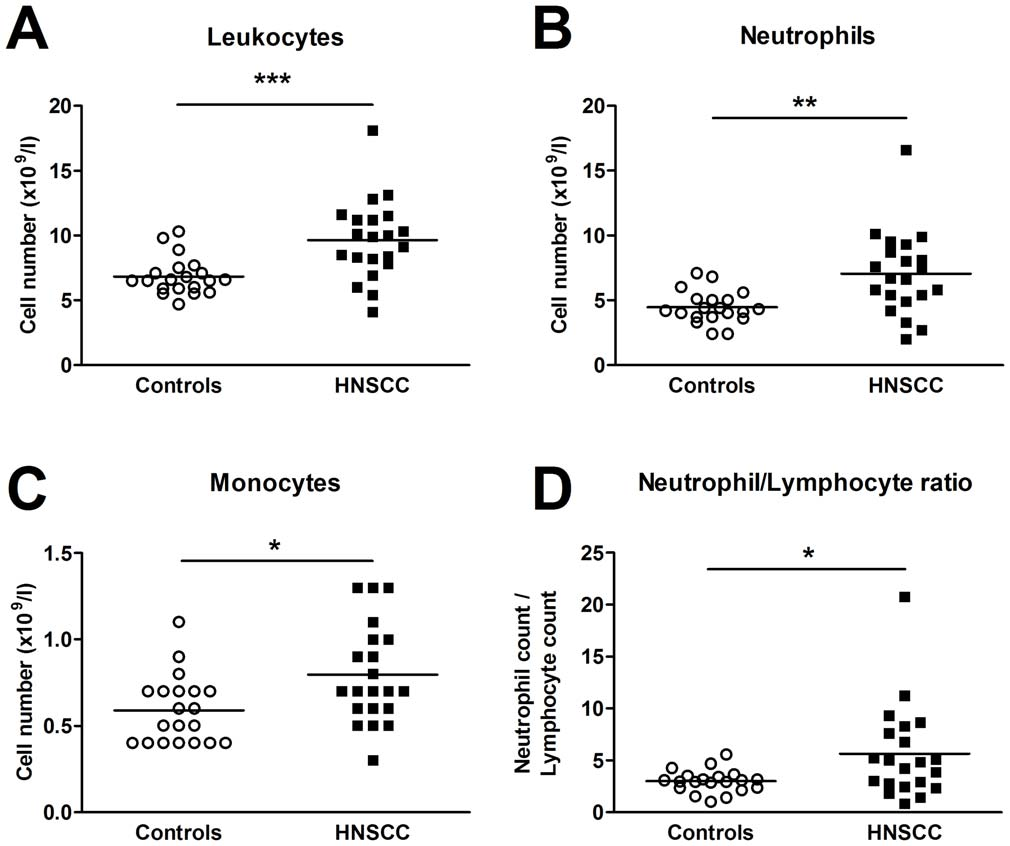
The number of (**A**) total leukocytes, (**B**) neutrophils, (**C**) monocytes, and (**D**) the neutrophil/lymphocyte ratio in blood from patients with HNSCC (*n* = 20) and controls subjects (*n* = 20) was determined by leukocyte differential count analysis. * *p*≤0.05; ** *p*≤0.01; *** *p*≤0.001.

A leukocyte differential count indicated that the total number of leukocytes, neutrophils and monocytes was higher in HNSCC patients than among controls (Fig. 1A–C). No such difference was observed for lymphocytes, eosinophils or basophils. The neutrophil/lymphocyte ratio, suggested to be an indicator of prognosis for various types of cancer [8], [9], [10], [11], was higher among the cancer patients (Fig. 1D). The percentage of various leukocyte subsets, CD3^+^ T cells, CD8^+^ cytotoxic T lymphocytes (CTLs), CD4^+^ Th cells (comprising both Th1 and Th2 cells), CD4^+^CRTH2^+^ Th2 cells and CD4^+^CD25^high^Foxp3^+^ regulatory T cells (Tregs), along with CD3^−^CD56^+^CD16^+^ NK cells, were analyzed using flow cytometry. In contrast to the differential count data, the flow cytometry analyses showed no change in the different leukocyte subsets between the HNSCC patients and the control individuals. Neither did the distribution of dendritic cells (DCs), as judged by staining of CD123^+^BDCA2^+^ plasmacytoid DCs (pDCs) and Lin^−^CD11c^+^HLA-DR^+^ myeloid DCs (mDCs), show any differences between the two groups analyzed (data not shown).

**Figure 2:**
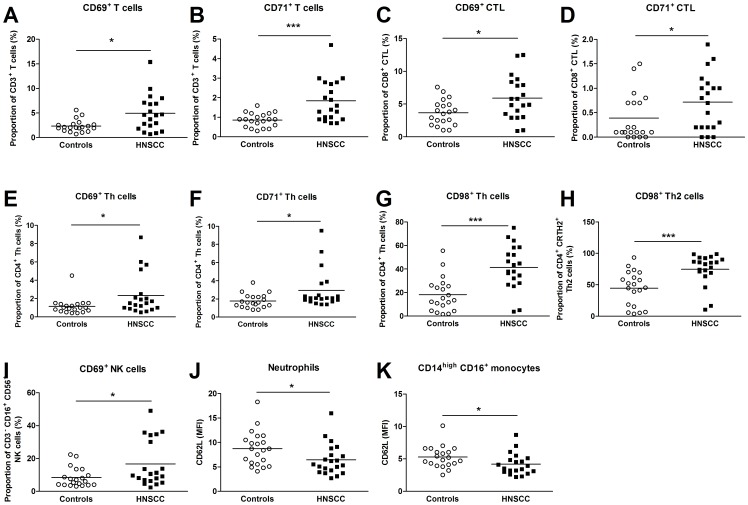
Blood from HNSCC patients (*n* = 20) and controls (*n* = 20) was incubated with different Abs and analyzed with flow cytometry. (**A, B**) Abs against CD3, CD69 and CD71 were used to distinguish activated total T cells. (**C, D**) To identify activated cytotoxic T lymphocytes (CTLs) CD69, CD71 and CD8 Abs were used. (**E, F, G**) CD4^+^ T helper (Th) cells were discriminated by CD4 Abs, and the activation status was established using CD69, CD71 and CD98 Abs. (**H**) Th2 cells were detected by CD4 and CRTH2 Abs, and the frequency of CD98^+^ Th2 cells was determined. (**I**) CD3, CD16, CD56 and CD69 Abs were used to discriminate CD69^+^ CD3^−^CD16^+^CD56^+^ natural killer (NK) cells. (**J**) Neutrophils were determined as CD16^+^ granulocytes, and the mean fluorescence intensity (MFI) of CD62L^+^ cells was calculated. (**K**) Staining of CD14, CD16 and CD62L was used to discriminate activation of the monocyte population CD14^high^CD16^+^. The MFI of CD62L^+^ CD14^high^CD16^+^ monocytes was established. * *p*≤0.05; ** *p*≤0.01; *** *p*≤0.001.

HNSCC patients had a higher value of the total T cell population positively stained for the early activation marker CD69 and the proliferation marker CD71 compared to healthy individuals (Fig. 2A and B). Further analyses of various T cell subsets revealed that an increased frequency of CTLs from patients with HNSCC expressed CD69 and CD71 (Fig. 2C and D) and Th cells from the same patients displayed an increased percentage of CD69, CD71 and CD98 (Fig. 2E–G). The CD98 upregulation might be related to the Th2 phenotype, as an increased number of Th2 cells was positive for CD98 (Fig. 2H). The Th presentation of the IL-2 receptor alpha-chain (CD25) did not differ between the two groups. Finally, the percentage of CD69^+^ NK cells was increased in cancer patients, whereas no difference was observed for the CD71^+^ NK cells (Fig. 2I and data not shown).

Neutrophils and monocytes, including various monocyte subpopulations (CD14^high^CD16^−^, CD14^high^CD16^+^ and CD14^dim^CD16^+^), were investigated for their expression of the adhesion molecule CD62L (L-selectin) and CD69. Neutrophils and CD14^high^CD16^+^ monocytes exhibited a low mean fluorescence intensity (MFI) of L-selectin (Fig. 2J, K) in HNSCC patients. No other discrepancies were observed.

**Figure 3:**
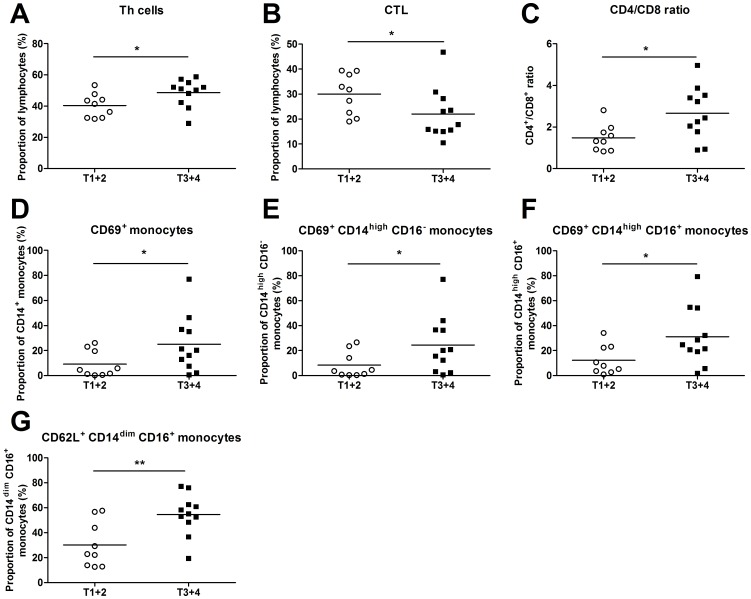
Blood from HNSCC patients was incubated with various Abs, followed by flow cytometry analysis. The HNSCC patients were divided according to the size of the primary tumor (stage T1/T2 *versus* T3/4; *n* = 9 and *n* = 11, respectively). (**A**) T helper (Th) cells were defined as CD4^+^ lymphocytes, whereas (**B**) cytotoxic T lymphocytes (CTLs) were identified by CD8, and (**C**) the CD4/CD8 ratio was calculated. (**D**) CD14 and CD69 were used to discriminate activated monocytes. (**E**) CD69 expressing CD14^high^CD16^−^ monocytes were identified. (**F**) The monocyte population CD14^high^CD16^+^ was recognized and the number of CD69^+^ cells was determined. (**G**) In addition, the percentage of CD62L^+^ CD14^high^CD16^+^ monocytes was established. * p≤0.05; ** p≤0.01.

**Figure 4:**
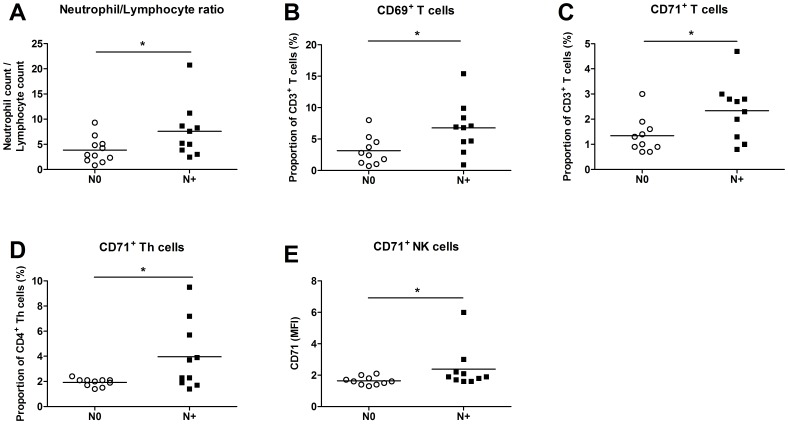
Blood was obtained from HNSCC patients and divided according to presence of regional lymphatic node metastases (N0 *versus* N+; *n* = 10 and *n* = 10, respectively). (**A**) The neutrophil/lymphocyte ratio was determined by leukocyte differential count analysis. (**B, C**) Blood was incubated with CD3, CD69 and CD71 Abs, and the percentage of activated total T cells was established with flow cytometry. (**D**) Abs against CD4 and CD71 were used to identify activated T helper (Th) cells. (**E**) The mean fluorescence intensity (MFI) of CD62L^+^ CD3^−^CD56^+^CD16^+^ natural killer (NK) cells was calculated. * *p*≤0.05.

When the size of the primary tumor, as either T1/T2 or T3/T4 (10), was related to the individual leukocyte activation pattern the T3/T4 group presented a higher number of Th cells and a lower percentage of CTL than the T1/T2 group (Fig. 3A and B). Accordingly, the CD4/CD8 ratio, often used as a prognostic indicator [12], [13], was higher among the T3/4 patients (Fig. 3C). In addition, the number of CD69 expressing monocytes as well as the CD14^high^CD16^−^ and CD14^high^CD16^+^ subgroups, was increased among those with a more advanced disease. This group had a higher frequency of CD14^dim^CD16^+^ monocytes positive for L-selectin (Fig. 3D–G). It is also worth noticing that patients with lymphatic spread (N+) exhibited a higher neutrophil/lymphocyte ratio than patients without metastases (N0). The N+ patients also displayed an elevated percentage of CD69^+^ and CD71^+^ T cells, a higher number of Th cells expressing CD71 and a higher CD71 density (MFI) on their NK cells (Fig. 4A–E).

**Figure 5:**
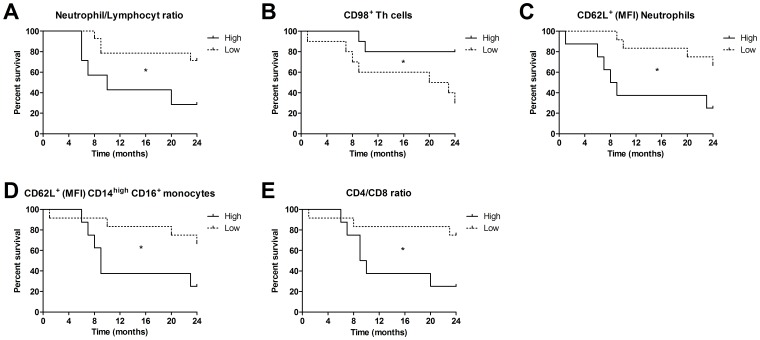
A Kaplan-Meier survival analysis following diagnosis of HNSCC patients, before start of treatment, appeared to be related to (**A**) the neutrophil/lymphocyte ratio, (**B**) expression of CD98 among the Th cells, (**C**) CD62L among neutrophils, and (**D**) CD14^high^ CD16^+^ monocytes, and (**E**) the CD4/CD8 ratio. The patients were divided according to the mean values of the parameters analyzed, characterized as either higher or lower than mean. * *p*≤0.05.

To evaluate the prognostic value of the leukocyte markers, described above, the patient's status at the time of cancer discovery, before any treatment was given, was related to the total survival during a 24 months observation period. The HNSCC group was divided according to the mean values of the different parameters, characterized as either higher than mean or lower than mean. The group with a high neutrophil/lymphocyte ratio had a worse outcome than the lower mean ratio group. A higher number of activated neutrophils and CD14^high^ CD16^+^ monocytes, determined by a lower CD62L expression, and an elevated number of CD98^+^ Th cells predicted a better survival (Fig. 5A–D). The CD4/CD8 ratio is an index with disputed prognostic value [3], [13], [14], [15], [16]. Here we show that patients with a high ratio were found to have a reduced survival (Fig. 5E). Generally, the prognostic value of the leukocyte factors seemed to be most pronounced during the first 12 months after diagnosis (Fig. 5A–E).

## Discussion

The present study suggests that the HNSCC patients display increased numbers of total leukocytes, neutrophils and monocytes, and accordingly a higher neutrophil/lymphocyte ratio than control subjects. The cancer patients also exhibit an enhanced percentage of activated T cell subsets and NK cells, as determined by an increase in the density of CD69, CD71 and CD98. In addition, CD14^high^CD16^+^ monocytes and neutrophils from HNSCC patients have a lower expression of L-selectin. The activation frequency of total T cells, Th cells, NK cells and monocyte populations seems to correlate with tumor burden and lymphatic spread in such a way that a high state of activation can be found among patients with a more severe disease. It also appears as if the neutrophil/lymphocyte ratio as well as the activation status of neutrophils, CD14^high^ CD16^+^ monocytes and Th cells, at the time of cancer diagnosis, is linked to the patient's life expectancy.

High numbers of neutrophils and monocytes were observed in HNSCC patients using differential count analysis. No such differences were found with flow cytometry. This disparity could be related entirely to the analysis methods used. It needs to be emphasized that classification of leukocytes by differential count depends on volume, conductivity and light scattering, whereas flow cytometry simply distinguishes leukocyte subsets by their cell surface markers. The difference in results may also be affected by the way data is presented as either absolute number or as percentage. In line with this, Kuss *et al.* has shown a reduced absolute number of total T cells, Th cells and CTLs in patients with HNSCC without any corresponding changes in the percentage [7].

The increased number of neutrophils and monocytes seen in the HNSCC patients are supported by previous reports showing similar findings in other types of cancer [8], [17], [18]. The increase might reflect an increased inflammation and an enhanced infiltration of immature neutrophils and monocytes from the bone marrow as a consequence of an increased leukocyte turnover. Accordingly, the high neutrophil-lymphocyte ratio, that was observed in the HNSCC patients, indicates an ongoing systemic inflammation [19]. Furthermore, a high neutrophil-lymphocyte ratio appears to be synonymous with a reduced survival rate, which suggests that a high systemic inflammation is linked to the patient's life expectancy. This is in accordance to recent reports suggesting this ratio to be a potential prognostic factor in several forms of cancer [8], [9], [10], [11], [19]. The role of neutrophils in cancer has recently gained attention. They are thought to be pro-tumorigenic by secretion of pro-angiogenic substances and suppression of the adaptive immune system [20], [21], [22]. In contrast, there are studies reporting an anti-tumorigenic role for these cells. Gregory and Houghton have suggested that activated neutrophils can elicit antitumor activity [22], [23]. In support of this, the present data demonstrates that HNSCC patients with a higher frequency of activated neutrophils have a better survival.

CD14^high^CD16^+^ monocytes are known to produce IL-10. The enhanced activation of these monocytes seen among the HNSCC patients can contribute to the elevated IL-10 concentration that has previously been found in serum of such patients [24], [25]. Therefore, it was no surprise that patients with an advanced disease exhibited a higher activation of these monocytes as compared to patients with a milder tumor burden. In contrast, the increased survival rate of patients with an elevated activation frequency of these IL-10 producing CD14^high^CD16^+^ monocytes was surprising. However, these monocytes might have until now unknown anti-tumor functions that might contribute to an increased survival. The present data indicate that, with the exception of the more mature monocyte population CD14^dim^CD16^+^, patients with an increased tumor burden generally exhibit a more active monocytes profile. It has previously been reported that there is an increased activation of immature macrophages in cancer patients [26]. This indicates that the activated monocytes in patients with a more advanced disease might be of a more immature phenotype.

The tumor appears to affect the frequency of CTLs and Th cells, as demonstrated by an increase in Th cells and a decrease in CTLs as well as a higher CD4/CD8 ratio, and thereby inhibiting the anti-tumorigenic CTL response and promoting a pro-tumorigenic environment. In accordance, previous reports have demonstrated an increase in the percentage of T cells and Th cells in addition to a decrease in CTLs in patients with an advanced tumor disease [4]. A high CD4/CD8 ratio was also connected to poor survival. The prognostic value of this ratio has been disputed by several studies showing contradictory results [3], [13], [14], [15], [16]. The present data supports the notion that this ratio indicates that patients with a high anti-tumorigenic response have a better chance of survival.

The high number of activated T cell subsets and NK cells among the HNSCC patients and specifically in patients with a verified lymph node metastasis reveals an enhanced immune activation that corresponds well with the increased neutrophil-lymphocyte ratio. Both indicate an accelerated systemic immune activity and an enhanced leukocyte turnover. In agreement, increased levels of CD69^+^ T cells have been shown in patients with advanced HNSCC [4]. The increase in Th2 activation might also be the result of the tumors to promote a pro-tumorigenic immune response. The increased activation of the Th2 cells was only accompanied by an increased expression of CD98 and not CD69 or CD71. It is not inconceivable that this observation reflects the fact that the microenvironment of the tumor attracts individual cells. The increased Th2 cell activation is well in analogy with previous studies reporting high levels of Th2 cytokines in serum, including IL-4, IL-6, IL-10 and granulocyte-monocyte colony-stimulating factor (GM-CSF) [1], [27]. The increased frequency of CD98^+^ Th cells that was presently found to be linked to survival could reflect an amplified Th1 anti-tumor activity, since no association was found for activated Th2 cells.

NK cells have cytolytic functions and are believed to play a role in tumor immune surveillance [28]. It is therefore tempting to speculate that the higher percentage of activated NK cells presently observed reflects an enhanced immune surveillance activity against the tumor. However, it must be considered that NK cells also have been attributed to impair anti-tumor functions in HNSCC [6], [29].

To conclude, the present study demonstrates that there is an increased systemic inflammation in HNSCC patients. This was for instance determined by an increased activation of leukocytes. It is also clear that a higher frequency of activation is found among patients with a more severe disease. Although, the number of patients investigated is limited and the cancer population is heterogeneous the results are clear cut indicating that the leukocyte activation state at the time of diagnosis can be of prognostic value for survival. Further, it signifies a tight link between the immune phenotype and the anti-tumor immune response and hence the survival of the patient.

## Supporting Information

Table S1 Antibody panel used for flow cytometry analysis. ^1^Th cells = T helper cells; ^2^CTLs = cytotoxic T lymphocytes; ^3^NK cells = natural killer cells; ^4^pDCs = plasmacytoid dendritic cells; ^5^mDCs = myeloid dendritic cells; ^6^Linage cocktail = CD3-, CD14-, CD16-, CD19-, CD20- and CD56-FITC.
(DOCX)

Figure S1 **Blood from HNSCC patients (n = 20) and controls (n = 20) was incubated with various Abs and analyzed with flow cytometry.** Lymphocytes, monocytes and granulocytes were distinguished based on FS and SS plotting. From these cell populations, CD3^+^ T cells, CD8^+^ cytotoxic T lymphocytes (CTLs), CD4^+^ T helper (Th) cells, CD4^+^CRTH2^+^ Th2 cells, CD3^−^CD56^+^CD16^+^ natural killer (NK) cells, CD16^+^ neutrophils, CD14^+^ monocytes, CD14^high^CD16^−^ monocytes, CD14^high^CD16^+^ monocytes and CD14^dim^CD16^+^ monocytes were discriminated. Staining of CD62L, CD69, CD71 and CD98 was used to determine the activation of the different leukocyte subsets.
(TIF)

## References

[1] Pries R, Nitsch S, Wollenberg B (2006) Role of cytokines in head and neck squamous cell carcinoma. Expert Rev Anticancer Ther 6: 1195–1203. 17020454 10.1586/14737140.6.9.1195

[2] Woods KV, El-Naggar A, Clayman GL, Grimm EA (1998) Variable expression of cytokines in human head and neck squamous cell carcinoma cell lines and consistent expression in surgical specimens. Cancer Res 58: 3132–3141. 9679981

[3] Bose A, Chakraborty T, Chakraborty K, Pal S, Baral R (2008) Dysregulation in immune functions is reflected in tumor cell cytotoxicity by peripheral blood mononuclear cells from head and neck squamous cell carcinoma patients. Cancer Immun 8: 10. 18547033 PMC2935775

[4] Aarstad HJ, Heimdal JH, Klementsen B, Olofsson J, Ulvestad E (2006) Presence of activated T lymphocytes in peripheral blood of head and neck squamous cell carcinoma patients predicts impaired prognosis. Acta Otolaryngol 126: 1326–1333. 17101596 10.1080/00016480600702092

[5] Heimdal JH, Aarstad HJ, Olofsson J (2000) Peripheral blood T-lymphocyte and monocyte function and survival in patients with head and neck carcinoma. Laryngoscope 110: 402–407. 10718427 10.1097/00005537-200003000-00013

[6] Xie L, Pries R, Kesselring R, Wulff S, Wollenberg B (2007) Head and neck cancer triggers the internalization of TLR3 in natural killer cells. Int J Mol Med 20: 493–499. 17786279

[7] Kuss I, Hathaway B, Ferris RL, Gooding W, Whiteside TL (2004) Decreased absolute counts of T lymphocyte subsets and their relation to disease in squamous cell carcinoma of the head and neck. Clin Cancer Res 10: 3755–3762. 15173082 10.1158/1078-0432.CCR-04-0054

[8] Cho H, Hur HW, Kim SW, Kim SH, Kim JH, et al (2009) Pre-treatment neutrophil to lymphocyte ratio is elevated in epithelial ovarian cancer and predicts survival after treatment. Cancer Immunol Immunother 58: 15–23. 18414853 10.1007/s00262-008-0516-3 PMC11029845

[9] Walsh SR, Cook EJ, Goulder F, Justin TA, Keeling NJ (2005) Neutrophil-lymphocyte ratio as a prognostic factor in colorectal cancer. J Surg Oncol 91: 181–184. 16118772 10.1002/jso.20329

[10] Halazun KJ, Aldoori A, Malik HZ, Al-Mukhtar A, Prasad KR, et al (2008) Elevated preoperative neutrophil to lymphocyte ratio predicts survival following hepatic resection for colorectal liver metastases. Eur J Surg Oncol 34: 55–60. 17448623 10.1016/j.ejso.2007.02.014

[11] Hirashima M, Higuchi S, Sakamoto K, Nishiyama T, Okada H (1998) The ratio of neutrophils to lymphocytes and the phenotypes of neutrophils in patients with early gastric cancer. J Cancer Res Clin Oncol 124: 329–334. 9692841 10.1007/s004320050178 PMC12200937

[12] Karlsson M, Lindberg K, Karlen P, Ost A, Thorn M, et al (2010) Evidence for immunosurveillance in intestinal premalignant lesions. Scand J Immunol 71: 362–368. 20500687 10.1111/j.1365-3083.2010.02377.x

[13] Giuntoli RL 2nd, Webb TJ, Zoso A, Rogers O, Diaz-Montes TP, et al (2009) Ovarian cancer-associated ascites demonstrates altered immune environment: implications for antitumor immunity. Anticancer Res 29: 2875–2884. 19661290

[14] da Silveira EJ, Miguel MC, Lima KC, Freitas Rde A, de Morais Mde L, et al (2010) Analysis of local immunity in squamous cell carcinoma of the tongue and lower lip. Exp Mol Pathol 88: 171–175. 19944682 10.1016/j.yexmp.2009.11.009

[15] Boucek J, Mrkvan T, Chovanec M, Kuchar M, Betka J, et al (2009) Regulatory T cells and their prognostic value for patients with Squamous Cell Carcinoma of the Head and Neck. J Cell Mol Med 10.1111/j.1582-4934.2008.00650.x PMC3837595 19183242

[16] Sheu BC, Hsu SM, Ho HN, Lin RH, Torng PL, et al (1999) Reversed CD4/CD8 ratios of tumor-infiltrating lymphocytes are correlated with the progression of human cervical carcinoma. Cancer 86: 1537–1543. 10526283 10.1002/(sici)1097-0142(19991015)86:8<1537::aid-cncr21>3.0.co;2-d

[17] Cho H, Kim JH (2009) Multiplication of neutrophil and monocyte counts (MNM) as an easily obtainable tumour marker for cervical cancer. Biomarkers 14: 161–170. 19399661 10.1080/13547500902777616

[18] Schmidt H, Bastholt L, Geertsen P, Christensen IJ, Larsen S, et al (2005) Elevated neutrophil and monocyte counts in peripheral blood are associated with poor survival in patients with metastatic melanoma: a prognostic model. Br J Cancer 93: 273–278. 16052222 10.1038/sj.bjc.6602702 PMC2361564

[19] Zahorec R (2001) Ratio of neutrophil to lymphocyte counts–rapid and simple parameter of systemic inflammation and stress in critically ill. Bratisl Lek Listy 102: 5–14. 11723675

[20] Koga Y, Matsuzaki A, Suminoe A, Hattori H, Hara T (2004) Neutrophil-derived TNF-related apoptosis-inducing ligand (TRAIL): a novel mechanism of antitumor effect by neutrophils. Cancer Res 64: 1037–1043. 14871835 10.1158/0008-5472.can-03-1808

[21] Kusumanto YH, Dam WA, Hospers GA, Meijer C, Mulder NH (2003) Platelets and granulocytes, in particular the neutrophils, form important compartments for circulating vascular endothelial growth factor. Angiogenesis 6: 283–287. 15166496 10.1023/B:AGEN.0000029415.62384.ba

[22] Fridlender ZG, Albelda SM (2012) Tumor-associated neutrophils: friend or foe? Carcinogenesis 33: 949–955. 22425643 10.1093/carcin/bgs123

[23] Gregory AD, Houghton AM (2011) Tumor-associated neutrophils: new targets for cancer therapy. Cancer Res 71: 2411–2416. 21427354 10.1158/0008-5472.CAN-10-2583

[24] Skrzeczynska-Moncznik J, Bzowska M, Loseke S, Grage-Griebenow E, Zembala M, et al (2008) Peripheral blood CD14high CD16+ monocytes are main producers of IL-10. Scand J Immunol 67: 152–159. 18201370 10.1111/j.1365-3083.2007.02051.x

[25] Heimdal JH, Aarstad HJ, Klementsen B, Olofsson J (1999) Ex vivo interleukin (IL)-1 beta, IL-6, IL-12 and tumor necrosis factor-alpha responsiveness with monocytes from patients with head and neck carcinoma. Eur Arch Otorhinolaryngol 256: 250–256. 10392301 10.1007/s004050050152

[26] Srivastava MK, Andersson A, Zhu L, Harris-White M, Lee JM, et al (2012) Myeloid suppressor cells and immune modulation in lung cancer. Immunotherapy 4: 291–304. 22401635 10.2217/imt.11.178 PMC3324285

[27] Lathers DM, Achille NJ, Young MR (2003) Incomplete Th2 skewing of cytokines in plasma of patients with squamous cell carcinoma of the head and neck. Hum Immunol 64: 1160–1166. 14630398 10.1016/j.humimm.2003.08.024

[28] Herberman RB, Ortaldo JR (1981) Natural killer cells: their roles in defenses against disease. Science 214: 24–30. 7025208 10.1126/science.7025208

[29] Bose A, Ghosh D, Pal S, Mukherjee KK, Biswas J, et al (2006) Interferon alpha2b augments suppressed immune functions in tobacco-related head and neck squamous cell carcinoma patients by modulating cytokine signaling. Oral Oncol 42: 161–171. 16249117 10.1016/j.oraloncology.2005.06.025

