# Supplementary material for: The Activation Pattern of Blood Leukocytes in Head and Neck Squamous Cell Carcinoma Is Correlated to Survival
Source: PLoS One. 2012 Dec 10;7(12):e51120. doi: 10.1371/journal.pone.0051120 (PMC3519486; doi:10.1371/journal.pone.0051120)
Supplement: Table S1 — Antibody panel used for flow cytometry analysis. 1Th cells = T helper cells; 2CTLs = cytotoxic T lymphocytes; 3NK cells = natural killer cells; 4pDCs = plasmacytoid dendritic cells; 5mDCs = myeloid dendritic cells; 6Linage cocktail = CD3-, CD14-, CD16-, CD19-, CD20- and CD56-FITC. (DOCX) [file pone.0051120.s001.docx]

**Table S1**

| **No.** | **Description** | **FL-1** | **FL-2** | **FL-3** | **FL-4** |
| --- | --- | --- | --- | --- | --- |
| I | Th cells^1^ | CD98 | CRTH2 | - | CD4 |
| II | Th cells | CD71 | CRTH2 | CD69 | CD4 |
| III | Tregs | Foxp3 | - | CD25 | CD4 |
| IV | CTLs^2^ | CD8 | - | CD69 | CD71 |
| V | T cells and NK cells^3^ | CD71 | CD69 | CD3 | CD56+CD16 |
| VI | Monocytes and granulocytes | - | CD62L | CD16 | CD14 |
| VII | Monocytes and granulocytes | CD14 | CD69 | CD16 | - |
| VIII | pDCs^4^ | CD123 | BDCA2 | - | - |
| IX | mDCs^5^ | Lineage cocktail^6^ | CD11c | - | HLA-DR |
| X | Isotype control | msIgG | msIgG1 | msIgG2b | msIgG1 |
