# Supplementary figures and images for: The Activation Pattern of Blood Leukocytes in Head and Neck Squamous Cell Carcinoma Is Correlated to Survival
Source: PLoS One. 2012 Dec 10;7(12):e51120. doi: 10.1371/journal.pone.0051120 (PMC3519486; doi:10.1371/journal.pone.0051120)

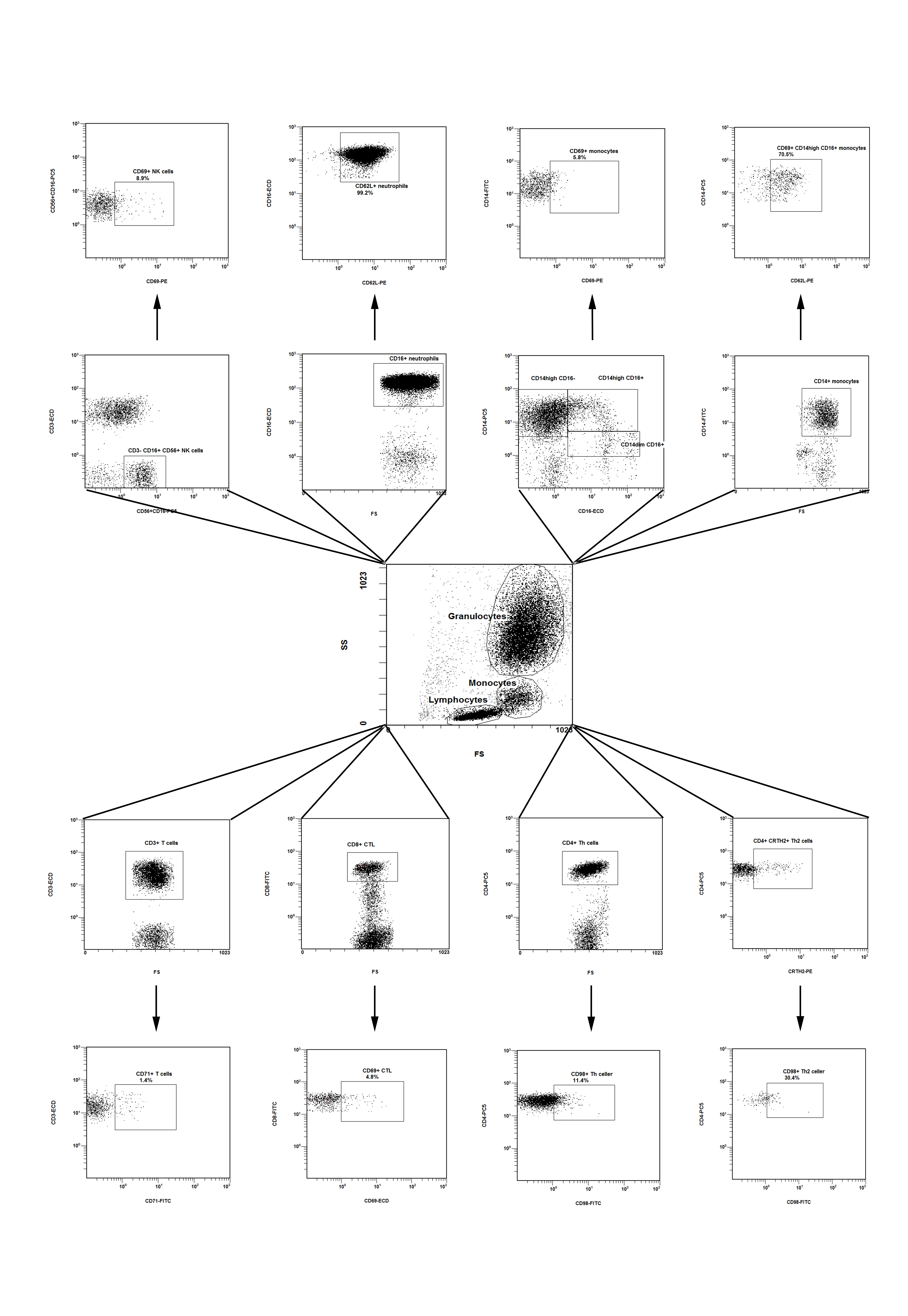

Supplement: Figure S1 — Blood from HNSCC patients (n = 20) and controls (n = 20) was incubated with various Abs and analyzed with flow cytometry. Lymphocytes, monocytes and granulocytes were distinguished based on FS and SS plotting. From these cell populations, CD3+ T cells, CD8+ cytotoxic T lymphocytes (CTLs), CD4+ T helper (Th) cells, CD4+CRTH2+ Th2 cells, CD3−CD56+CD16+ natural killer (NK) cells, CD16+ neutrophils, CD14+ monocytes, CD14highCD16− monocytes, CD14highCD16+ monocytes and CD14dimCD16+ monocytes were discriminated. Staining of CD62L, CD69, CD71 and CD98 was used to determine the activation of the different leukocyte subsets. (TIF) [file pone.0051120.s002.tif]
